# Genomic surveillance of HBV and HDV reveals genotype-specific risk of liver disease in central Vietnam

**DOI:** 10.1038/s41598-025-31423-1

**Published:** 2025-12-10

**Authors:** Le Chi Cao, Tran Thi Tien Xinh, Dang Ngoc Phuoc, Nguyen Thi Dung, Tran Thi Kim Loan, Pham Van Duc, Dao Thi Huyen, Le Thi Kieu Linh, Le Huu Song, Thirumalaisamy P. Velavan

**Affiliations:** 1 Institute of Tropical Medicine, University of Tübingen, and German Center for Infection Research (DZIF), Wilhelmstrasse 27, Tübingen, 72074 Germany; 2 Hue University of Medicine and Pharmacy (HUMP), Hue University, Hue, 49000 Vietnam; 3 Vietnamese-German Center for Medical Research (VG-CARE), Hanoi, 10000 Vietnam; 4 108 Military Central Hospital, Hanoi, Vietnam; 5 Faculty of Medicine, Duy Tan University, Danang, 550000 Vietnam

**Keywords:** Hepatitis B, Hepatitis D, RT mutation, Hepatocellular carcinoma, Liver cirrhosis, Diseases, Gastroenterology, Genetics, Microbiology, Molecular biology

## Abstract

Hepatitis B virus (HBV) genotype distribution, resistance mutations, and hepatitis delta virus (HDV) co-infection play critical roles in disease progression and treatment outcomes yet remain understudied in central Vietnam. This study aimed to understand their characteristics and association with clinical outcomes. Serum from 376 HBV-infected patients in Hue, Vietnam (June 2023-June 2024) was analysed. HBV-DNA and HDV-RNA were extracted and amplified by nested PCR, followed by Sanger sequencing. HBV viral load was quantified using real-time PCR. HBV and HDV genotypes were determined through phylogenetic analysis, and HBV resistance mutation identified using geno2pheno [hbv]. HBV genotype B was predominant (95%), followed by genotypes C (5%) and D (0.3%). Infection with genotype C was significantly associated with an increased risk of hepatocellular carcinoma (OR = 6.05, 95% CI: 1.6–22.5; *p* = 0.007). Classical drug resistance mutations were rare (1.3%), while non-classical mutations were observed in 33% of sequences, especially V207M in genotype B. HDV co-infection was identified in two patients (0.5%), both infected with HDV genotype 1 and associated with elevated liver enzymes and liver disease progression. In conclusion, HBV genotype C emerged as a key predictor of hepatocellular carcinoma risk in Central Vietnam, highlighting the need of genotype-based monitoring. Low antiviral resistance and HDV co-infection likely reflect effective vaccination and treatment in the region. Routine HDV screening and resistance surveillance are recommended.

**Supplementary Information:**

The online version contains supplementary material available at 10.1038/s41598-025-31423-1.

## Introduction

Hepatitis B virus (HBV) continues to be a major health burden, infecting an estimated 250 million individuals and causing approximately 80,000 deaths annually, due to complications such as HBV-related cirrhosis and hepatocellular carcinoma (HCC)^1^. In Southeast Asia, and specifically in Vietnam, the burden is even more pronounced, with an estimated 10% of the population chronically infected with HBV^2^. Notably, nearly 90% of HCC cases in Vietnam are associated with viral hepatitis, predominantly HBV (60%) and hepatitis C virus (HCV) (30%)^3^. This underscores the urgent need for comprehensive and timely strategies to reduce the burden of HBV infection in the general population.

HBV genotyping is essential especially in endemic regions like Vietnam, where the prevalence of specific HBV genotypes influences disease progression and treatment outcomes. At least ten HBV genotypes (A-J) and over 40 subgenotypes have been identified, with distinct geographical distributions^4^. Genotype C, known for its higher virulence, is associated with severe liver disease, including cirrhosis and HCC. The distribution of genotype C in Vietnam varies from 14% to 31%^5,6^, highlighting the need for genotype-specific management strategies to improve patient outcomes. The rising number of antiviral treatment failures in Vietnam, particularly with lamivudine (LAM), telbivudine (LDT) and entecavir (ETV) is concerning. These failures are often associated with resistance mutations, such as M204I/V, L180M/Y/R/F, A181T/G and L80I^6,7^, which compromise the effectiveness of standard therapies. This trend emphasizes the urgent need for continuous monitoring of HBV mutation profiles in the Vietnamese population to guide treatment decisions and reduce the risk of therapy failure.

Hepatitis delta virus (HDV) is a defective satellite virus that depends on the hepatitis B surface antigen (HBsAg) for replication. Co-infection with HDV accelerates disease progression in patients with chronic hepatitis B (CHB), significantly increasing the risk of cirrhosis and HCC^8^. In Vietnam, the prevalence of HDV among HBV infected individuals varies widely, ranging from 1% to 16%^5,9,10^, However, a nationwide HDV screening program has not yet been widely implemented, which may hinder timely diagnosis and intervention^2^. Of the eight HDV genotypes (HDV-1 to HDV-8) identified thus far, HDV-1 predominates in Vietnam, is associated with higher rates of cirrhosis and HCC compared to other HDV genotypes^5,9^. Therefore, genotyping of HDV in HBV-infected individuals in Vietnamese population is critical for optimizing HBV treatment strategies and informing national HDV screening policies.

In this context, this study aims to investigate the circulation of HBV genotypes in relation to clinical outcomes, resistance mutations, and the prevalence of HDV co-infection. By exploring these factors, the study aims to provide critical insights that could guide more effective management and control strategies for HBV in Vietnam, particularly through genotype-specific treatment approaches and the implementation of routine HDV screening.

## Results

### Demographic data and clinical characteristics

The characteristics of HBV patients are summarized in Table 1. The cohort included both male and female patients (ratio male/female = 209/164), aged from 8 to 97 years, with a diverse representation of socioeconomic backgrounds (Table 1). Among the CHB cohort, the majority (*n* = 312) had no evidence of advanced liver disease, as defined by clinical, biochemical, and imaging criteria. The other CHB patients were diagnosed with liver cirrhosis (CHB + LC; *n* = 42), with hepatocellular carcinoma (CHB + HCC; *n* = 10) and with both cirrhosis and HCC (CHB + LC + HCC; *n* = 9) confirmed by clinical assessment and imaging studies. Significant differences were observed between the patient groups in terms of age, white blood cell count (WBC), red blood cell count (RBC), hemoglobin (Hb), platelet count (PLT), aspartate aminotransferase (AST), alanine aminotransferase (ALT), prothrombin time (PT), albumin and alpha-fetoprotein (AFP) (all *p* < 0.001; Table 1). However, no statistically significant differences were found in HBV viral load between the AHB, CHB, CHB + LC, CHB + HCC and CHB + LC + HCC groups (*p* > 0.05; Table 1), suggesting that viral load alone may not be a reliable indicator of liver disease severity in this cohort. In this study, 241 patients (72%) were receiving antiviral therapy, with the highest treatment rate observed in the CHB + LC + HCC group (89%). There was no significant difference in treatment rates among the other subgroups (Table 1).

**Table 1:** Characteristics HBV patients segregated according to clinical presentation and healthy controls.

	Hepatitis B infection (*n* = 376)				
	CHB (no LC and HCC) (*n* = 312)	CHB + LC (*n* = 42)	CHB + HCC (*n* = 10)	CHB + LC + HCC (*n* = 9)	AHB (*n* = 3)
Age (years)*	46 [8–97]	58 [26–78]	68 [40–73]	59 [29–79]	34 [21–43]
Sex ratio (M/F)	167/145	25/17	9/1	8/1	3/0
WBC (10^3^/µl)*	6.8 [3.3–20.0]	5.1 [2.7–11.5]	7.1 [5.1–11.1]	6.7 [4.2–19.4]	7.8 [5.7–8.6]
RBC (10^6^/µl)*	4.6 [2.8–7.2]	4.1 [2.6–5.2]	4.4 [3.0–5.5.0.5]	4.3 [2.9–4.6]	4.3 [3.9–4.5]
Hb (g/L)*	140 [63–184]	127 [91–166]	144 [94–170]	130 [93–153]	133 [117–152]
PLT (10^3^/µl)*	214 [60–597]	102 [39–324]	221 [123–508]	150 [64–239]	129 [64–188]
AST (IU/L)*	27 [12–308]	59 [20–737]	42 [24–72]	72 [51–659]	405 [344–1504]
ALT (IU/L)*	27 [6–305]	53 [16–517]	39 [10–88]	87 [21–741]	949 [716–2053]
Prothrombin Time (s)*	12.5 [9.3–38.4]	15.3 [3.1–29.5]	13.8 [11.1–17.1]	14.0 [12.7–23.4]	15.9 [10.9–18.6]
Albumin (g/L)*	43.1 [11.4–50.9]	34 [19–46.6.6]	39.7 [36.6–44.6]	31.5 [28.5–41.6]	43.8
AFP (ng/mL)*	2.6 [0.9–23.2]	15 [1–238]	40.5 [3–29136]	864 [3–10053]	87.1
HBV viral loads (copies/mL)	3.2 × 10^3^ [221 − 3.2 × 10^9^]	3.6 × 10^3^ [221 − 4.1 × 10^8^]	7.1 × 10^4^ [284 − 1.2 × 10^7^]	9.7 × 10^3^ [284 − 6.2 × 10^6^]	1.5 × 10^6^ [4.1 × 10^5^ − 2.8 × 10^6^]
Under anti-viral treatment (%)	195/312 (63%)	32/42 (76%)	6/10 (60%)	8/9 (89%)	0/3 (0%)

CHB: Chronic hepatitis B; LC: liver cirrhosis; HCC: hepatocellular carcinoma; AHB: Acute hepatitis B; WBC: white blood cells; RBC: red blood cells; PLT: platelets; AST and ALT: aspartate and alanine amino transferase; AFP: alpha-feto protein; IU: international unit; Values are presented as medians and ranges. *p < 0.001 for comparisons with all other groups using a one-way ANOVA test.

### HBV genotyping and clinical outcomes

**Fig. 1:**
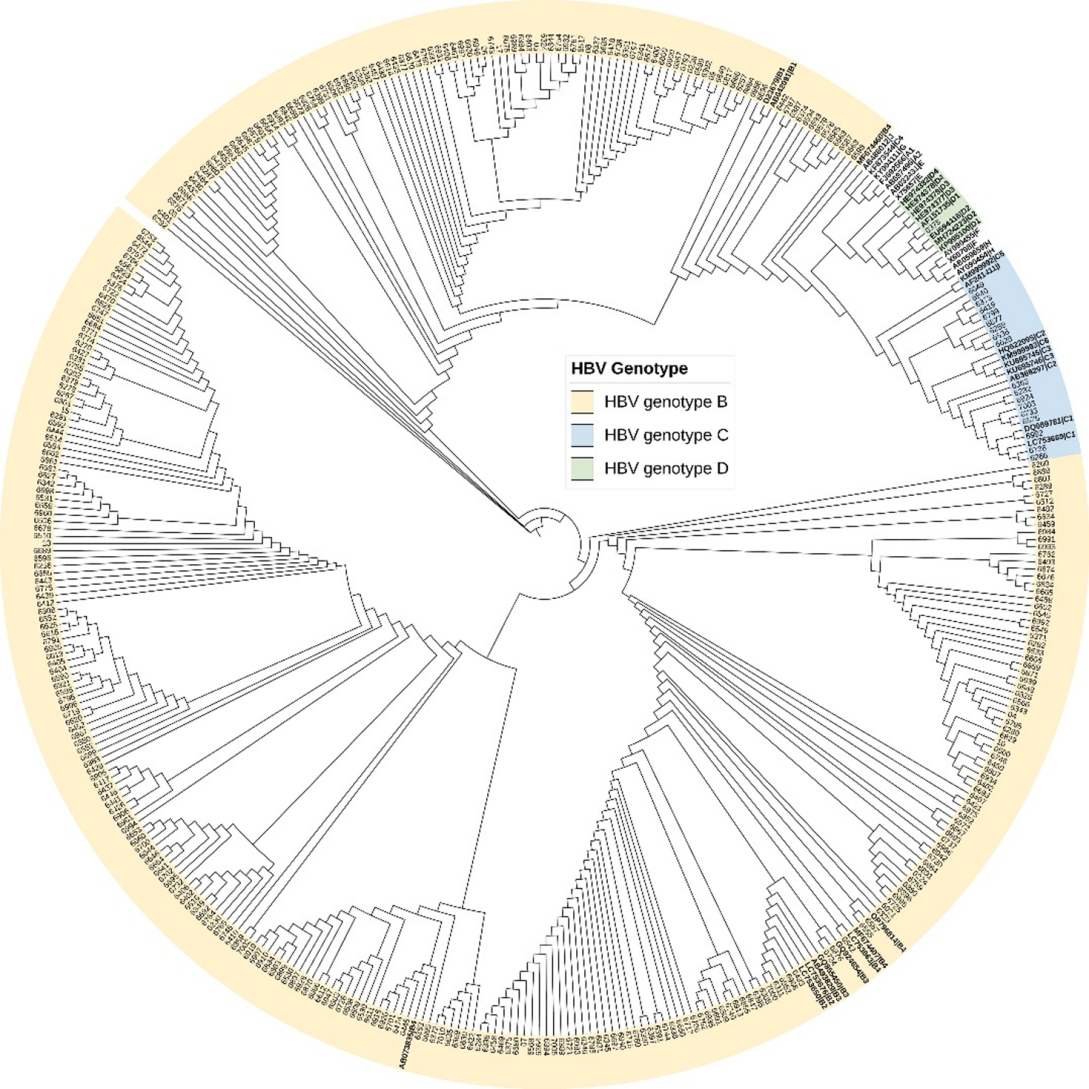
Phylogenetic tree of HBV S/P gene sequences. The tree was constructed from 376 sequences obtained in this study and 40 reference sequences (genotypes A–J, shown in bold). Study sequences cluster within genotypes B, C, and D.

All 376 HBV-positive samples were successfully sequenced. Phylogenetic analysis revealed that most sequences belonged to genotype B (95%, 357/376), followed by genotype C (5%, 18/376), with a single sequence classified as genotype D (0.3%, 1/376) (Fig. 1). Genotype B, common in Southeast Asia, was predominant and is typically associated with a higher likelihood of developing chronic infection. When stratified by clinical outcomes, genotype C was identified in 4% of CHB patients without LC or HCC, but its prevalence was higher among patients with CHB + HCC (10%) and CHB + LC + HCC (33%) (Table 2). To assess the association between genotype C and progression to LC or HCC relative to genotype B, binary logistic regression was performed adjusting for age and sex and anti-viral treatment status. Genotype C was associated with a sixfold increased risk of HCC compared to genotype B (OR = 6.05, 95% CI: 1.6–22.5; *p* = 0.007) (Table 3). Post-hoc power (two-sided, α = 0.05): 61.3%. Although genotype C also appeared more frequently in patients with LC, the association was not statistically significant (OR = 2.4, 95% CI: 0.7–6.9, *p* = 0.141) (Table 3), indicating that genotype C may play a more prominent role in the progression to HCC.

**Table 2:** Distribution of HBV genotypes and clinical outcomes.

HBV Patients	Genotype B - *n* (%)	Genotype C - *n* (%)	Genotype D - *n* (%)
CHB (*n* = 312)	300 (96)	12 (4)	0
CHB + LC (*n* = 43)	40 (93)	2 (5)	1 (2)
CHB + HCC (*n* = 10)	9 (90)	1 (10)	0
CHB + LC + HCC (*n* = 9)	6 (66)	3 (33)	0
Acute HBV (*n* = 3)	3 (100)	0	0
Total (*n* = 376)	357 (95)	18 (5)	1 (0.2)

CHB: chronic hepatitis B; LC: liver cirrhosis; HCC: hepatocellular carcinoma.

**Table 3:** HBV genotypes and risk of cirrhosis and HCC.

HBV genotype	CHB vs. LC		CHB vs. HCC	
	OR (95% CI)	p*-* value	OR (95% CI)	p-value
Genotype B (*n* = 357)	Reference	0.141	Reference	**0.007**
Genotype C (*n* = 18)	2.4 (0.7–6.9)		6.05 (1.6–22.5)	

CHB: chronic hepatitis B; LC: liver cirrhosis; HCC: hepatocellular carcinoma; OR: Odd Ratio. Adjusted P values were calculated using binary logistic regression model adjusted for age and gender and antiviral treatment.

### Distribution of resistant mutation sites with different HBV genotypes

**Fig. 2:**
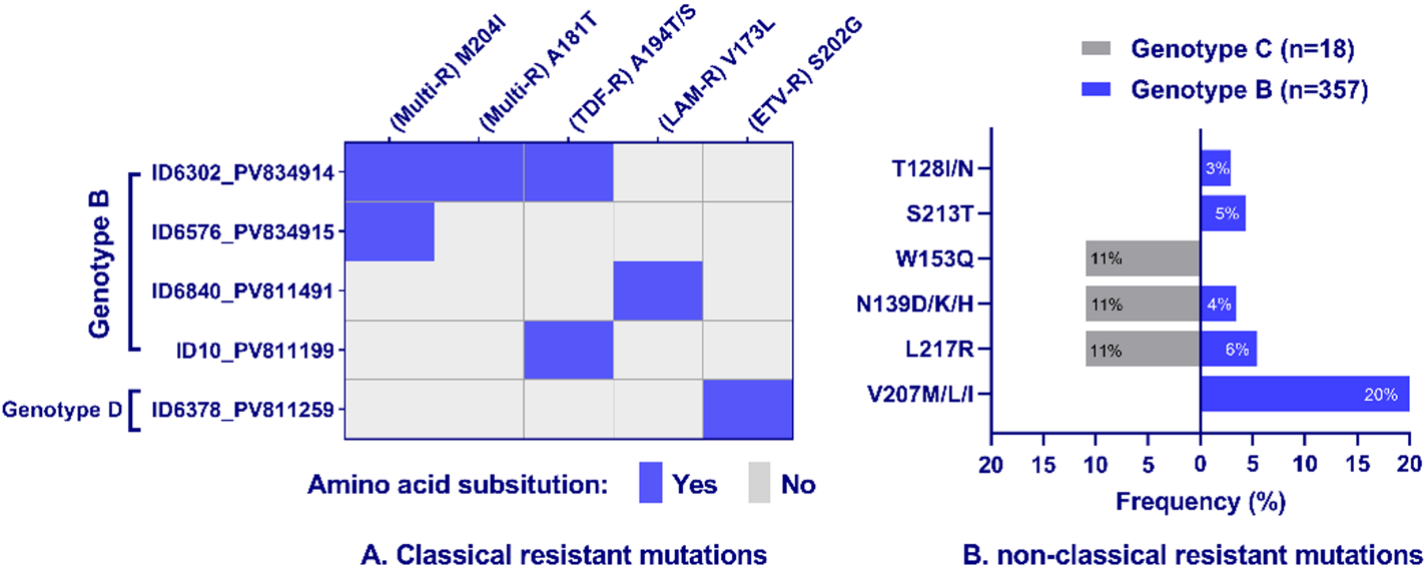
Distribution of HBV antiviral resistance mutations in the reverse transcriptase (RT) domain. Analysis of 376 sequences revealed **(A)** classical resistance mutations with established in vitro phenotypes and **(B)** non-classical mutations with potential resistance roles. Abbreviations: R, resistant; TDF, tenofovir; LAM, lamivudine; ETV, entecavir.

All 376 sequences were analyzed for drug resistance mutations within the RT domain. Classical resistance mutations were identified only in five sequences (1.3%) (Fig. 2A). Among these, one sample (ID6302) harbored three co-occurring mutations: M204I, A181T, and A194T. The remaining four samples each contained a single classical mutation: M204I, A194S, V173L, or S202G (Fig. 2A). Resistance mutations in these samples are of clinical interest, as they can compromise the effectiveness of antiviral therapies like lamivudine and entecavir. Three patients (ID6302, ID6576, ID6840) had favorable clinical outcomes, with no evidence of HCC or LC, suggesting that other factors, such as treatment adherence and host immune response, may influence disease progression. In contrast patient ID10 who carried the A194S mutation, developed both LC and HCC, and patient ID6378 (genotype D, S202G) also progressed to LC. Detailed clinical and virological characteristics of these five patients are provided in Supplementary Table S1.

Analysis of non-classical mutations across all 376 sequences revealed that 125 sequences (33%) harbored at least one mutation site, including 119 sequences from genotype B (33%), 5 sequences from genotype C (28%), and 1 sequence from genotype D. In genotype B (*n* = 357), the most prevalent mutation was V207M/L/I, detected in 73 sequences (20%). Other mutations, including L217R, S213T, N139D/K/H, and T128I/N, were observed at lower frequencies (3–6%, Fig. 2B). These mutations are of particular interest as they may affect antiviral resistance, although their clinical significance is still under investigation. In genotype C (*n* = 18), three mutations, including L217R, N139D/K/H, and W153Q were observed with each present in 2 sequences (11%). W153Q was found exclusively in genotype C (Fig. 2B). The sequence of genotype D (ID6378) carried a non-classical mutation N139H, which may be associated with reduced treatment efficacy.

### HDV co-infection and genotyping in HBV-infected patients

**Fig. 3:**
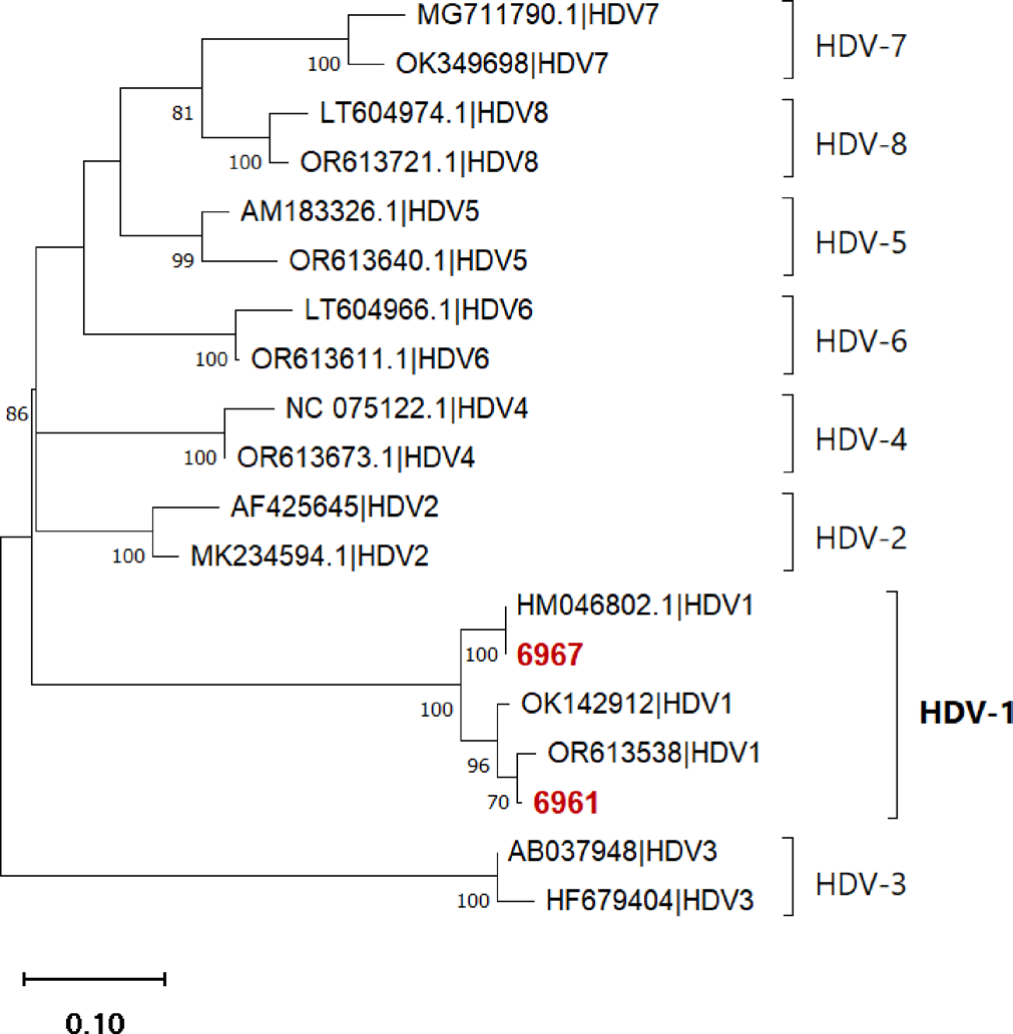
Phylogenetic tree of HDV antigen conserved regions. The tree includes two sequences from this study (#6967 and #6961), which cluster within genotype HDV-1, together with 17 reference sequences representing HDV genotypes 1–8.

HDV nested PCR screening of 376 HBV patient samples detected HDV-RNA in 2 cases (0.5%). Phylogenetic analysis showed both HDV positive cases belonged to HDV genotype 1 (Fig. 3). Although the prevalence of HDV co-infection was low in this cohort, its clinical significance is important. Patient ID6961 (CHB with LC) had markedly elevated AST and ALT levels (737 IU/L and 512 IU/L), while patient ID6967 (CHB without LC or HCC) showed moderate increases in AST and ALT (61 U/L and 94 U/L). Both patients were infected with HBV genotype B and had high HBV viral loads of approximately 1 × 10⁶ and 7 × 10⁸ copies/mL, respectively. The presence of HDV in these patients suggests that routine HDV screening should be considered in HBV patients, particularly those with cirrhosis.

## Discussion

Globally, HBV genotypes display distinct geographic distributions, which are influenced by regional transmission routes. HBV Genotypes A and D are predominant in Europe and Africa, whereas genotypes B and C are most prevalent in Asia, where perinatal or vertical transmission is common. In contrast, other genotypes, including A and D, are more commonly associated with horizontal transmission in low-endemic areas^11,12^. These geographic and transmission patterns are crucial for understanding the epidemiology of HBV and tailoring region-specific interventions. In Southeast Asia, the relative prevalence of genotypes B and C varies geographically. For example, genotype B predominates in Malaysia (57%), and Indonesia (56%), while genotype C is more prevalent in Thailand (85%), Myanmar (67%), and Cambodia (71%)^4,13^. In Vietnam, previous studies have reported a predominance of genotype B (ranging from 65 to 85%) and a lower prevalence of genotype C (10–30%), with only sporadic reports of genotype D, G and I^4,5,14^. In our study, genotype B remained dominant (95%), with a lower prevalence of genotype C (5%), which is consistent with regional patterns observed in central Vietnam^15^, in contrast to higher frequencies (31%) reported in northern Vietnam^5^. The lower frequency of HBV genotype C in our study may be influenced by limited inclusion of CHB patients with advanced liver disease, conditions more commonly associated with genotype C^1^.

Our study observed a significantly higher proportion of genotype C in patients with HCC (27%) compared to those with chronic HBV infection alone or with liver cirrhosis (4–5%). Multivariate logistic regression revealed that individuals infected with genotype C had an almost sixfold increased risk of developing HCC compared to those with genotype B, even after adjusting for age, sex, and antiviral treatment. This finding is consistent with previous reports suggesting that genotype C has a higher oncogenic potential^5,16^. Genotype C is considered the most oncogenic HBV genotype, characterized by delayed HBeAg seroconversion, prolonged viremia, and increased HBV DNA integration^17^. These features contribute to persistent inflammation, increased liver fibrosis, and ultimately an increased risk of HCC. In addition, a higher prevalence of basal core promoter (BCP) mutations observed in genotype C has been shown to further enhance the risk of HCC^16^. These observation reinforce the clinical significance of genotype C as a predictor of poor prognosis, emphasizing the need for genotype-specific treatment and monitoring strategies in Vietnam.

Additionally, our study found that age and male sex were significantly associated with the development of cirrhosis and HCC, consistent with previous report^18^. While the number of genotype C cases in our cohort was relatively small, post-hoc power analysis indicated approximately 61% power to detect the observed association (adjusted OR = 6.05), suggesting that the effect is robust but imprecisely estimated. Based on our data, approximately 28 genotype C patients would be needed to achieve 80% power at this effect size. Therefore, our findings support genotype C as an independent predictor of HCC risk but also highlight the need for larger, multicenter studies to refine risk estimates and confirm these results in more diverse populations.

In terms of drug resistance, analysis of resistance mutations in the RT domain revealed a low prevalence (1.3%) of classical mutations in our cohort. This contrasts with previous studies in Vietnam, which reported much higher rates, ranging from 33% in treatment-naïve patients to 100% in treatment-failure cases^6,7^. Common mutations such as M204I/V, L180M, A181V/T are associated with resistance to LAM, ETV, and LDT^7,19^. In our study, we observed one sample (ID6302) with three co-occurring mutations: M204I, A181T, A194T, which are associated with multi-drug resistance^20^. Despite the presence of these mutations, the patient responded well to TDF treatment, with a low viral load, HBeAg negativity, and no signs of LC and HCC. Interestingly, these mutations may have resulted from G-to-A hypermutation, a process driven by APOBEC3 cytidine deaminases, a host antiviral defense. This process can generate resistance mutations while impairing HBV replication by introducing stop codons or deleterious amino acid changes^21^. Tenofovir (TDF) was incorporated into Vietnam’s Social Health Insurance scheme in November 2020^22^, and has since become the preferred antiviral therapy owing to its superior efficacy and higher genetic barrier to resistance compared with older agents such as lamivudine (LAM)^23^. The low prevalence of classic resistance mutations observed in this study likely reflects the combined effects of improved national treatment guidelines and expanded health insurance coverage in Vietnam. Since the introduction of TDF as first-line therapy in the updated national HBV treatment guidelines and its inclusion in the national health insurance programme in 2020, access to and adherence to antiviral therapy have improved significantly. These developments have led to a reduction in treatment interruptions and minimized the selection pressure that favors the development of resistance. Therefore, the observed decline in antiviral resistance is likely due to both the successful implementation of national treatment recommendations in favor of antiviral drugs with a high genetic barrier and improved treatment continuity supported by national health insurance.

Moreover, our study identified non-classical resistance mutations, which are putative mutations not yet experimentally confirmed but likely relevant to NA resistance. Mutations such as V207M/L/I, S213T, T128I/N, and W153Q, were more frequently observed in genotype B, aligning with previous findings that reported a high prevalence of V207M in Vietnam^6^. These mutations, though not yet experimentally verified, are crucial for future investigations into resistance patterns, especially in treatment-naïve populations. Additionally, we found the L217R mutation, which may be associated with reduced response to adefovir, present in both genotypes B and C. While a Chinese study suggested that genotype C harbors more single-base mutations than genotype B^24^, our analysis showed no significant difference in resistance mutations between the two genotypes (B and C) in this cohort, indicating that both genotypes may share similar patterns of resistance to treatment.

Regarding HDV co-infection, our study found a notably low prevalence (0.5%) of HDV in chronic HBV patients, which is significantly lower than (13–16%) previous reports from Vietnam^5,9,25^. This finding may reflect regional or cohort differences, as well as the positive impact of improved HBV vaccination coverage and strengthened infection control efforts in Vietnam. The three-dose hepatitis B immunization program introduced in 1997 has reduced HBV prevalence to below 1% among children within the following decade^26^. The low prevalence of HDV (0.5%) observed in this study likely reflects multiple factors. The expansion of Vietnam’s national HBV vaccination program^27^ has reduced the reservoir of chronic HBV carriers and thereby lowered opportunities for HDV transmission. Regional variation may also play a role, with higher HDV rates reported in northern provinces and among high-risk populations. Our cohort consisted mainly of outpatients from central Vietnam without major HDV risk factors, and detection may have been influenced by sampling period, geography, and assay sensitivity. Additionally, recent public-health initiatives promoting early HBV screening among blood donors, healthcare workers, and surgical patients have enabled earlier diagnosis and treatment, reducing the likelihood of HDV co- or superinfection. In earlier years, HBV infection was often detected only at advanced stages of liver disease, when HDV superinfection risk was higher.

In our study, both HDV-positive cases were identified as HDV genotype 1 and were associated with elevated liver enzymes and high HBV viral loads. One of these patients had developed cirrhosis. However, given the very small sample size, it is not possible to establish a causal relationship between HDV infection and advanced liver disease. The global estimate of HDV co-infection among HBV carriers is around 13%^8^, and while the prevalence of HDV co-infection in our cohort was low, its presence in patients with cirrhosis underscores the importance of routine HDV screening, particularly among those with advanced liver disease.

In conclusion, this study demonstrates the predominance of HBV genotype B in central Vietnam, which is associated with more favorable clinical outcomes, while genotype C is associated with an increased risk of HCC. The low prevalence of classical resistance mutations and HDV co-infection may reflect the success of national antiviral treatment programs and widespread HBV vaccination efforts. However, several limitations should be noted. The cross-sectional design limits causal inference between genotypes, resistance mutations, and clinical outcomes. Additionally, the small number of genotype C and HDV-positive cases may reduce the statistical power of subgroup analyses. Furthermore, incomplete treatment histories and the lack of longitudinal follow-up data may hinder a full understanding of the long-term effects of HBV genotypes and resistance mutations on disease progression.

## Materials and methods

### Ethic statement

This study received approval from the Ethics Committee of Hue University of Medicine and Pharmacy, Hue University, Vietnam (H2024/585). Written informed consent was obtained from all participants. All experimental procedures were conducted in accordance with relevant ethical guidelines and regulations.

### Study design and collection

Blood samples were collected from 376 individuals diagnosed with HBV infection at the Hospital of Hue University of Medicine and Pharmacy, Central Vietnam, between June 2023 and June 2024. All patients were residing in Central Vietnam at the time of sampling. Given that Vietnam is a highly endemic country where HBV is predominantly transmitted from mother to child and adult infections are overwhelmingly acquired locally, these cases can be considered representative of autochthonous HBV infections. Patients were eligible for inclusion if they were HBsAg-positive or had a documented history of HBV infection with ongoing antiviral treatment. Patients without evidence of current HBV infection (negative by both quantitative PCR and HBV nested PCR), as well as those who declined participation, were excluded.

Participants were classified into two groups: acute hepatitis B (AHB, *n* = 3) and chronic hepatitis B (CHB, *n* = 373). AHB was defined by HBsAg positivity for less than 6 months, elevated liver enzymes (more than five times the upper limit of normal), and positive anti-HBc IgM. CHB was characterized by persistent HBsAg positivity for more than 6 months. Among the CHB cohort, 42 patients were diagnosed with liver cirrhosis (CHB + LC), 10 developed hepatocellular carcinoma (CHB + HCC), and 9 had both cirrhosis and HCC (CHB + LC + HCC). Cirrhosis was diagnosed based on clinical symptoms, biochemical abnormalities, and ultrasonographic evidence of chronic liver parenchymal damage. HCC diagnosis was confirmed through clinical manifestations, imaging studies, and histopathological examination.

### Nucleic acid isolation

Nucleic acid was isolated from serum samples using the QIAamp Viral DNA Mini Kit for HBV DNA extraction (Qiagen GmbH, Hilden, Germany) and the QIAamp Viral RNA Mini Kit for HDV RNA extraction (Qiagen GmbH, Hilden, Germany) according to the manufacturer’s instructions. The quality and quantity of nucleic acids were assessed using NanoDrop™ (Thermo Fisher Scientific, Waltham, MA, USA). Isolated nucleic acids were stored at −80 °C until further analysis.

### HBV quantification by real-time PCR

HBV viral load was quantified using real-time PCR targeting a conserved 90-bp region of the S gene. The SensiFAST™ one step RT-PCR kit (Meridian Biosciences, Memphis, Tennessee, USA) was used with a LightCycler 480 system (Roche Diagnostics, Rotkreuz, Switzerland). Each reaction was set up in a total volume of 20 µL, including 0.8 µL each of 10 µM forward primer (HBV-61) and reverse primer (HBV-62), 10 µL of 2× RT-PCR mix, 0.3 µL of probe (HBV-ITM-05) (Supplementary Table S2), 3.1 µL of water, and 5 µL of DNA template (15–20 ng). The cycling conditions consisted of an initial denaturation at 95 °C for 5 min, followed by 45 cycles of 95 °C for 10 s and 60 °C for 34 s. The assay had a detection limit of 25 IU/mL, calibrated using a plasmid standard (10⁶ copies/µL) with tenfold serial dilutions. Cycle threshold (Ct) values were used to calculate viral load, with a maximum Ct of 40 corresponding to 221 copies/mL.

### HBV qualitative nested PCR

The presence of HBV DNA in serum samples was confirmed using a nested PCR assay targeting a highly conserved region of the HBV genome (S/P region, 332 bp), as previously described^5^. PCR amplification was performed in a 25 µL reaction mixture containing 1× PCR buffer, 0.2 mM dNTPs, 0.4 µM of each specific primer (Eurofins Genomics, Ebersberg, Germany), and 1U of Taq DNA Polymerase (Qiagen GmbH, Hilden, Germany). For the outer PCR, primers HBV-022, HBV-65 and HBV-66 were used. The inner PCR utilized primers HBV-24, HBV-41, and HBV-64 (Supplementary Table S2). The thermal cycling conditions for the outer PCR included an initial denaturation at 95 °C for 15 min, followed by 35 cycles of denaturation at 94 °C for 30 s, annealing at 55 °C for 30 s, and extension at 72 °C for 30 s and a final extension at 72 °C for 5 min. The inner PCR followed the same cycling conditions, except the annealing temperature was 54 °C. Each PCR included both positive and negative controls to ensure the validity of the results. Amplicons were analyzed by electrophoresis on a 1.5% agarose gels stained with SYBR Green.

### RT-PCR for HDV-RNA detection

HDV RNA was reverse transcribed into cDNA using the High-Capacity cDNA Reverse Transcription Kit (Thermo Fisher Scientific, Waltham, MA, USA). HDV RNA was detected using nested PCR with primers targeting the conserved regions of the HDV antigen segment^5^. The outer PCR used primers HDV-04 and HDV-05, while the inner PCR utilized primers HDV-06 and HDV-07 (Supplementary Table S2). PCR amplification was carried out in a 15 µL reaction mixture containing 5 ng of cDNA, 1× PCR buffer, 0.2 mM dNTPs, 0.4 µM of each specific primer (Eurofins Genomics, Ebersberg, Germany), and 1 U of Taq DNA Polymerase (Qiagen GmbH, Hilden, Germany). Thermal cycling conditions for both the outer and nested PCR included an initial denaturation at 94 °C for 5 min, followed by 35 cycles of denaturation at 94 °C for 30 s, annealing at 54 °C (outer) or 58 °C (nested) for 30 s, and extension at 72 °C for 30 s. A final extension was performed at 72 °C for 5 min. A plasmid containing HDV cDNA served as a positive control. PCR products (235 bp) were analyzed by electrophoresis on 1.5% agarose gels stained with SYBR Green.

### Sanger sequencing and phylogenetic analysis

Nested PCR-positive samples for HBV and HDV were purified using ExoSAP-IT PCR (Thermo Fisher Scientific, Waltham, MA, USA) and sequenced with the BigDye™ Terminator v.3.1 Cycle Sequencing Kit (Thermo Fisher Scientific, Waltham, MA, USA) on an Applied Biosystems 3130xl Genetic Analyzer (Applied Biosystems, Beverly, MA, USA). The sequences were curated by combining the forward and reverse reads and assembling them using SeqMan version 6.1 (DNASTAR, Lasergene, USA). Consensus sequences of the HBV samples were aligned with 40 reference sequences representing HBV genotypes and subgenotypes A-H, retrieved from the NCBI Genotyping Tool (https://ncbi.nlm.nih.gov/projects/genotyping/), using MAFFT version 7^28^. Similarly, the sequence from the HDV-positive sample was aligned with 17 reference sequences representing HDV genotypes 1–8 obtained from the NCBI GenBank database (https://www.ncbi.nlm.nih.gov/datasets/genome/?taxon=12475). Phylogenetic trees were constructed using the neighbor-joining method with the Kimura 2-parameter model plus gamma distribution (K2 + G) and 1,000 bootstrap replicates in MEGA11^29^. The resulting trees were visualized and annotated in iTOL^30^. All sequences have been deposited in the NCBI database under accession numbers PV811192-PV811565 and PV834914-PV834915 for HBV, and PV811190-PV811191 for HDV.

Nucleos(t)ide analogue (NA) resistance mutations in the HBV polymerase gene were analyzed using the geno2pheno tool (https://hbv.geno2pheno.org). The amplicon covered the reverse transcriptase (RT) domain (amino acids 110–219) of the HBV polymerase gene. Classical resistance mutations, including M204I/V, L180M, A181T/V/S, T184A/G/S/I, A194T/S, S202G/I, and M204I/V, were examined. Non-classical mutations, such as Y126C, T128I/N, N139D/K/H, W153Q, F166L, A200V/P, V207M/I, S213T, V214A, Q215S, and L217R, were also evaluated^20,31,32^.

### Statistical analysis

Statistical analyses were performed using GraphPad Prism (v9.5.1) for univariate analyses and SPSS (v20) for multivariate analyses. Quantitative data were expressed as medians with ranges, and categorical data as counts and percentages. Chi-square or Fisher’s exact tests were used for categorical variables, and t-tests or Kruskal-Wallis tests for continuous variables. *P*-values < 0.05 were considered statistically significant.

## Supplementary Information

Below is the link to the electronic supplementary material.

Supplementary Material 1

## Data Availability

All sequences have been deposited in the NCBI database (https://www.ncbi.nlm.nih.gov/nucleotide/) under accession numbers PV811192-PV811565 and PV834914-PV834915 for HBV, and PV811190-PV811191 for HDV.
