# Supplementary material for: Genomic surveillance of HBV and HDV reveals genotype-specific risk of liver disease in central Vietnam
Source: Sci Rep. 2025 Dec 10;15:43491. doi: 10.1038/s41598-025-31423-1 (PMC12695881; doi:10.1038/s41598-025-31423-1)
Supplement: Supplementary file 1 — Supplementary Material 1 [file 41598_2025_31423_MOESM1_ESM.docx]

**Supplementary Table S1.** Clinical and virological characteristics of five patients harboring classical HBV resistance mutations.

| **No** | **Patient ID** | **Age** | **Gender** | **Viral load**  **(copies/mL)** | **HBeAg status** | **HBV genotype** | **Mutation rtHBV** | **Current treatment** | **HBV outcomes** |
| --- | --- | --- | --- | --- | --- | --- | --- | --- | --- |
| 1 | ID6302 | 39 | Male | 2.84 × 10^2^ | Negative | Genotype B | M204I, A181T, A194T | TDF | CHB |
| 2 | ID6576 | 72 | Male | Undetectable | Negative | Genotype B | M204I | TDF | CHB |
| 3 | ID6840 | 36 | Male | 4.04 × 10^6^ | Positive | Genotype B | V173L | Not yet | CHB |
| 4 | ID10 | 79 | Male | 2.84 × 10^2^ | Negative | Genotype B | A194S | Not yet | CHB+LC+HCC |
| 5 | ID6378 | 47 | Female | 2.84 × 10^2^ | Positive | Genotype D | S202G | TDF | CHB+LC |
| TDF: Tenofovir, CHB: Chronic hepatitis B, LC: Liver cirrhosis, HCC: Hepatocellular carcinoma | | | | | | | | | |

**Supplementary Table S2.** The detail of Oligonucleotide used in the study

| **Primer** | **Oligonucleotide** | **Reference** |
| --- | --- | --- |
| **HBV quantification by real-time PCR** | | |
| HBV-61 | 5′-GGACCCCTGCTCGTGTTACA-3′ | [Mbencho MN et al.](https://journals.plos.org/plosone/article?id=10.1371/journal.pone.0312126) |
| HBV-62 | 5′-GAGAGAAGTCCACCACGAGTCTAGA-3′ |  |
| HBV-ITM-05 | FAM-5′-TGTTGACAARAATCCTCACAATACCRCAGA-3′-DabCyl |  |
| **HBV Qualitative nested PCR** | | |
| HBV-022 | 5′-TGCTGCTATGCCTCATCTTC-3′ | [Hoan NX et al.](https://www.mdpi.com/1999-4915/13/2/346) |
| HBV-65 | 5′-CAAAGACAAAAGAAAATTGG-3′ |  |
| HBV-66 | 5′-CACAGATAACAAAAAATTGG-3′ |  |
| HBV-24 | 5′-CAAGGTATGTTGCCCGTTTGTCCT-3′ |  |
| HBV-41 | 5′-GGACTCAMGATGYTGCACAG-3′ |  |
| HBV-64 | 5′-GGACTCACGATGCTGTACAG-3′ |  |
| **HDV-RNA detection using RT-PCR** | | |
| HDV-04 | 5'-GAGGCYATGGTSGAGAARG-3' | [Hoan NX et al.](https://www.mdpi.com/1999-4915/13/2/346) |
| HDV-05 | 5'-AAGAAGAGRAGCCGGCCCGY-3' |  |
| HDV-06 | 5'-ATGCCATGCCGACCCGAAGA-3' |  |
| HDV-07 | 5'-GGGGAGCGCCCGGDGGCGG-3' |  |
